# Ajulemic acid: potential treatment for chronic inflammation

**DOI:** 10.1002/prp2.394

**Published:** 2018-04-06

**Authors:** Sumner H. Burstein

**Affiliations:** ^1^ Department of Biochemistry & Molecular Pharmacology University of Massachusetts Medical School Worcester MA USA

**Keywords:** CB2, inflammation‐resolving, disease‐modifying, antimetastatic, systemic sclerosis

## Abstract

Ajulemic acid (AJA, CT‐3, IP‐751, JBT‐101, anabasum) is a first‐in‐class, synthetic, orally active, cannabinoid‐derived drug that preferentially binds to the CB2 receptor and is nonpsychoactive. In preclinical studies, and in Phase 1 and 2 clinical trials, AJA showed a favorable safety, tolerability, and pharmacokinetic profile. It also demonstrated significant efficacy in preclinical models of inflammation and fibrosis. It suppresses tissue scarring and stimulates endogenous eicosanoids that resolve chronic inflammation and fibrosis without causing immunosuppression. AJA is currently being developed for use in 4 separate but related indications including systemic sclerosis (SSc), cystic fibrosis, dermatomyositis (DM), and systemic lupus erythematosus. Phase 2 clinical trials in the first 3 targets demonstrated that it is safe, is a potential treatment for these orphan diseases and appears to be a potent inflammation‐resolving drug with a unique mechanism of action, distinct from the nonsteroidal anti‐inflammatory drug (NSAID), and will be useful for treating a wide range of chronic inflammatory diseases. It may be considered to be a disease‐modifying drug unlike most NSAIDs that only provide symptomatic relief. AJA is currently being evaluated in 24‐month open‐label extension studies in SSc and in skin‐predominant DM. A Phase 3 multicenter trial to demonstrate safety and efficacy in SSc has recently been initiated.

**Abbreviations**
AJA: ajulemic acid
AUC: area under the curve
BAL: bronchoalveolar lavage
BLM: bleomycin
BrdU: 5‐bromo‐2‐deoxyuridine
CF: cystic fibrosis
CFTR: cystic fibrosis transmembrane conductance regulator
CFUs: colony forming units
CNS: central nervous system
CRISS: Combined Response Index in Diffuse Cutaneous Systemic Sclerosis
CYP1A2: cytochrome P450 isoform 1A2
CYP2C19: cytochrome P450 isoform 2C19
CYP2C9: cytochrome P450 isoform 2C9
CYP2D6: cytochrome P450 isoform 2D6
CYP3A4/5: cytochrome P450 isoform 3A4/5
DM: dermatomyositis
EAE: experimental autoimmune encephalomyelitis
LOX: lipoxygenase
LXA_4_: lipoxin A_4_
MS: multiple sclerosis
NSAID: nonsteroidal anti‐inflammatory drug
PA: *Pseudomonas aeruginosa*
PGD_2_: prostaglandin D_2_
PGJ: 15‐deoxy‐Δ^12,14^‐prostaglandin‐J_2_
RA: rheumatoid arthritis
SM: sulfur mustard
SSc: systemic sclerosis
THC: tetrahydrocannabinol
TLRs: toll‐like receptors
USAMRICD: U.S. Army Medical Research Institute of Chemical Defense
WBCs: white blood cell counts
WT: wild type

## 1 INTRODUCTION

**Figure 1:**
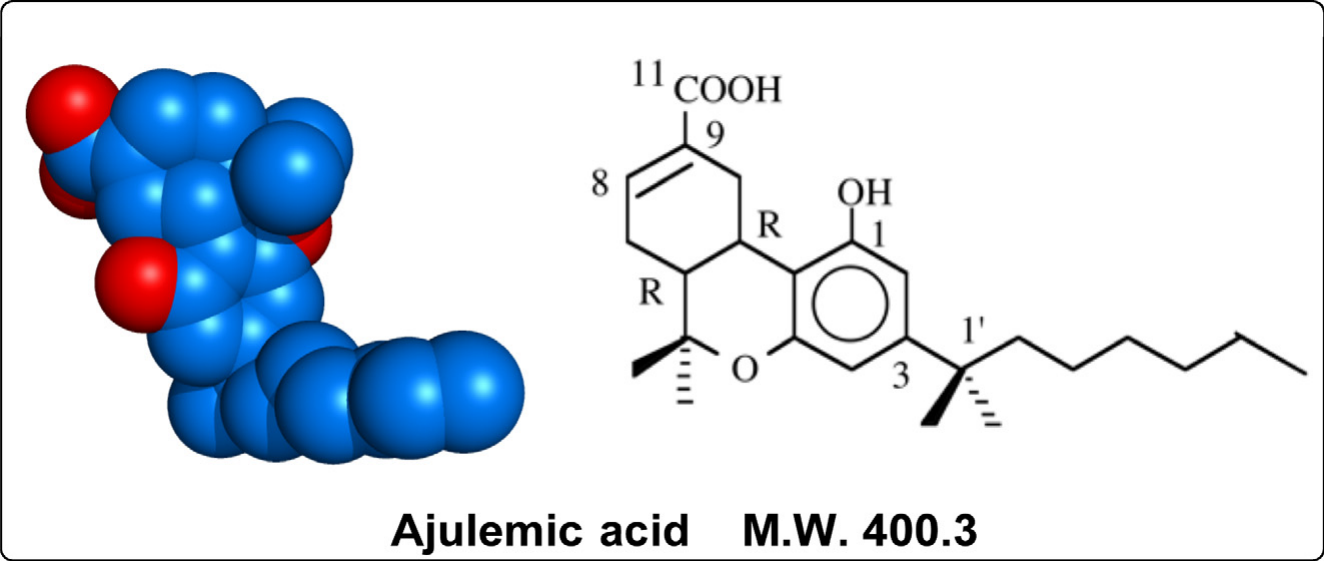
The structure of ajulemic acid. (6a*R*,10a*R*)‐1‐hydroxy‐6,6‐dimethyl‐3‐(2‐methyloctan‐2‐yl)‐6a,7,10,10a‐tetrahydrobenzo[c]chromene‐9‐carboxylic acid. Molecular formula: C_25_H_36_O_4_. Exact mass: 400.261 g/mol. It is asynthetically modified analog of THC‐11‐oic acid, the principal metabolite of THC. THC, tetrahydrocannabinol

The chemical structure of ajulemic acid (AJA) is shown in Figure 1. It is a synthetic analog of THC‐11‐oic acid, the principle metabolite of tetrahydrocannabinol (THC), the mood‐altering component of *Cannabis*. AJA, unlike THC, does not produce behavioral changes either in animals or in humans; however, it does retain several of the therapeutically useful actions of THC. A recent review describes in a concise manner the nature of these activities.^1^ This review will focus on the remarkable progress that has been reported since the previous review, mainly in the clinical development of AJA as an inflammation‐resolving agent. In addition, several of its potentially important preclinical actions are now reviewed in some detail.

## 2 BACKGROUND AND HISTORY

The preclinical literature on AJA has been briefly reviewed,^2^ however, in this presentation, several selected, as well as newly reported areas, are discussed in more detail. These have been chosen because they suggest therapeutic targets where AJA may be efficacious in the treatment of human disease. Specifically, they are arthritis, fibrosis and metastatic disease. Each of the 3 represent a major under met medical need.

### 2.1 Rheumatoid arthritis

Joint tissue injury in patients with rheumatoid arthritis (RA) is due in part to activation of T lymphocytes in the synovia; T lymphocytes in synovia of RA patients are resistant to apoptosis. Oral administration of AJA prevents joint cartilage and bone damage in an experimental model of arthritis in rats.^3^ Thus, a potential mechanism whereby AJA prevents joint tissue injury in this animal model might be an enhanced apoptosis of T lymphocytes. Apoptosis of human T cells in vitro was assessed by annexin V expression, caspase‐3 activity, DNA fragmentation, and microscopy.^4^, ^5^ AJA induced apoptosis of T cells in a dose‐ and time‐dependent manner. Apoptosis preceded loss of cell viability by trypan blue dye exclusion, confirming that cell loss was due to programmed death rather than necrosis. This suggests that a nontoxic compound such as AJA may be a useful therapeutic agent for patients with diseases such as RA that are characterized by T‐cell‐driven chronic inflammation and tissue injury. Reduced T‐cell proliferation was promoted by the addition of AJA to cells 60 minutes before stimulation with anti‐CD3/CD4 monoclonal antibodies. AJA suppressed 5‐bromo‐2‐deoxyuridine (BrdU) incorporation in a concentration‐dependent manner; half‐maximal inhibition was achieved with concentrations lower than 1 μmol/L.

Because activation of osteoclasts is essential in the pathogenesis of bone erosion in patients with RA, the influence of AJA on osteoclast differentiation and survival was studied. Osteoclast cultures were established by stimulation of RAW264.7 (RAW) cells, and primary mouse bone marrow cultures, with the receptor activator of NF‐kappa B ligand (RANKL).^5^ Simultaneous addition of AJA (15 or 30 μmol/L) and RANKL to both culture systems significantly suppressed development of multinucleated osteoclasts (osteoclastogenesis) in a dose‐dependent manner, as determined by quantification of multinuclear, tartrate‐resistant, acid phosphatase‐positive cells. AJA impaired growth of RAW264.7 monocytes and prevented further osteoclast formation in cultures in which osteoclastogenesis had already begun. Reduction by AJA of both monocyte growth and osteoclast formation was associated with apoptosis, assayed by annexin V, propidium iodide staining, and caspase activity. The antiosteoclastogenic effects of AJA did not require the continuous presence of AJA in the cell cultures. Based on these findings, it seems likely that AJA may be a useful drug for diseases such as RA and osteoporosis in which bone resorption is a central feature.

### 2.2 Multiple sclerosis

In multiple sclerosis (MS), damage to myelin in the central nervous system (CNS) and to the nerve fibers themselves interferes with the transmission of nerve signals between the brain, the spinal cord, and other parts of the body. The loss of myelin promotes several inflammatory processes, which results in the release of soluble factors like cytokines and antibodies that further perpetuates nerve damage. Data were obtained in a mouse model of experimental autoimmune encephalomyelitis (EAE) at doses of 0.1 and 1.0 mg/kg AJA. It was identified as a substance that is able to reduce MS‐induced spasticity presumably by its inflammation resolving action on peripheral nerves.^6^

### 2.3 Antifibrotic effects

Bleomycin (BLM)‐induced experimental fibrosis in mice was used to assess the antifibrotic effects of AJA in vivo.^7^, ^8^ Initially, acute inflammation characterized by neutrophil and macrophage accumulation were the main changes present in lung parenchyma.^8^ Between 8 and 14 days after BLM administration, the response progresses from inflammation to fibrosis and involves the bronchi and vasculature. The fibrotic response in lung tissue at day 21 after BLM treatment was significantly reduced in mice receiving either AJA in the inflammatory or in the early fibrogenic phase. A marked change in the expression pattern of substances implicated in fibrogenesis, such as TGF‐β1, pSMAD2/3, CTGF, and α‐SMA was observed.

### 2.4 Cancer and antimetastatic effects

Early observations on inhibition of cell proliferation by AJA (Burstein SH and Zurier RB, unpublished) prompted a study of its possible anticancer actions.^9^ AJA was compared with THC as an antineoplastic agent and was found to be nearly equipotent in vitro and more effective than THC in vivo. For example, at 14 days following inoculation of glioma cells, tumor diameter was 50% less in AJA‐treated mice when compared to vehicle or THC‐treated animals. Data obtained with the CB2 receptor antagonist SR144528 suggested that CB2 was involved in the mechanism of the antitumor action.

It has been proposed that inflammation can be promoted by endogenous mediators through Toll‐like receptors (TLRs) to enhance tumor progression and metastasis.^10^ Many studies have shown a correlation between TLR signaling and tumor progression and metastasis; endogenous mediators, including HMGB1 (high mobility group box 1), have been implicated in the triggering of tumor‐associated inflammation. A gene array study showed that AJA significantly reduced the expression of TLR 3, 5, 7, 8, and 9 (Skulas A and Zurier RB, personal communication) suggesting a role for these receptors in the antimetastatic effects of AJA.

Inflammation in the tumor microenvironment that is mediated by IL‐1β is believed to have an important role in cancer invasiveness, progression, and metastases.^11^ Thus, further support for the anticancer action of AJA comes from the report that it can inhibit the production of IL‐1β.^12^ An example of such a mechanism comes from a randomized, double‐blind, placebo‐controlled trial of the IL‐1β inhibitor, canakinumab.^13^ Thus, it is quite reasonable to suggest that IL‐1β inhibitors, such as AJA, could produce a similar therapeutic action without side effects.

**Figure 2:**
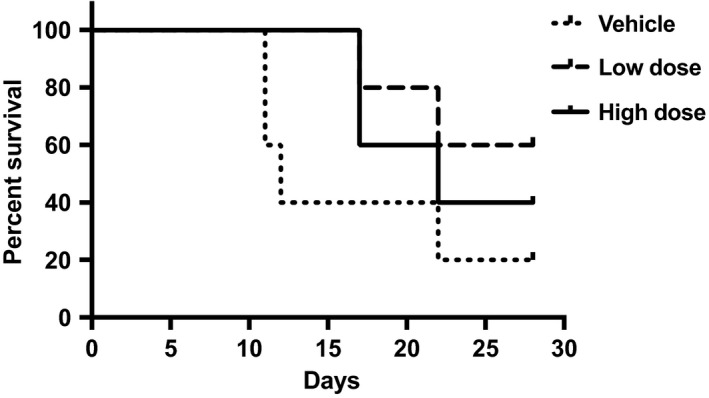
Ajulemic acid increases survival time in an inverse dose‐response relationship in a mouse glioma model (Recht L and Salmonsen R, unpublished). High dose, 10 mg/kg; low dose, 0.1 mg/kg. AJA administration began 1 day after U87MG cell inoculation into the right flank of SCID‐NOD mice. N = 5/group. Calculated using Prism and the method of Kaplan and Meier. AJA, ajulemic acid

Based on the earlier encouraging findings, a study of the effect of AJA on survival time was initiated (Recht L and Salmonsen R, personal communication). SCID‐NOD mice (n = 5/group) were inoculated subcutaneously with glioma cells and the course of the cancer was followed up for 28 days. The survival data are shown in Figure 2. These limited data suggest a modest improvement in survival with the lower dose being more effective. Of greater interest is the possible reduction of metastatic disease in the AJA‐treated mice as evidenced by the absence of ascites and a better overall condition of the AJA‐treated mice. This may be partly due to the anti‐inflammatory properties of AJA and to its effect on several matrix metalloproteinases.^14^ Also, the appearance of the primary tumors was different in the drug‐treated mice vs. the vehicle‐treated mice. The AJA‐treated mice had discrete well‐defined tumors, whereas the vehicle‐treated control mice produced diffuse, poorly defined tumors; the latter is typical of the human clinical situation and is a major problem in the treatment of glial cell brain cancer. This suggests that AJA, with its good safety profile, would be a useful follow up long‐term treatment to suppress the development of metastatic disease.

### 2.5 A medical countermeasure against nerve and blister agents

Inflammatory cytokines and proteolysis are implicated in the toxicity of both the blister agent sulfur mustard (SM [2,2′‐dichlorodiethyl sulfide]) and the nerve agent soman.^15^ Synthetic analogs of THC can have potent anti‐inflammatory effects that involve inhibition of cytokine activity without significant psychotropic action.^2^ The ability of AJA to reduce the increased inflammatory cytokines IL‐8 and TNF in SM‐exposed human epidermal keratinocyte cultures was evaluated by the U.S. Army Medical Research Institute of Chemical Defense (USAMRICD (Atlantic Pharmaceuticals, unpublished)). AJA dose dependently suppressed IL‐8 and TNF in HD‐exposed human epidermal keratinocyte cultures; AJA was added 1 hour following SM treatment. SM‐induced increases in both cytokines were completely inhibited at a 10 μmol/L concentration of AJA. Treatment of HD‐exposed human epidermal keratinocyte cultures with AJA also improved morphological changes associated with SM toxicity observed by phase‐contrast microscopy. Chemical warfare dates back to 1000 BC with the use of arsenical smokes by the Chinese. Recent events in the Middle East have renewed the need for effective treatment of injuries resulting from exposure to nerve and blister agents. The actions of AJA described above suggest its use as one possibility.

### 2.6 Pathogen resolution comparing wild type with CFTR‐deficient mice; a model for CF

Pulmonary infection and inflammation are major factors in the morbidity and mortality in CF. A study was performed to determine the safety of AJA in a murine model of chronic *Pseudomonas aeruginosa* (PA) lung infection and inflammation, and to establish the therapeutic potential of AJA in CF lung infection using chronically infected CFTR‐deficient mice.^16^ In the first series of studies, wild‐type (WT) C57BL/6J animals were utilized to evaluate oral dosing, safety and toxicity of AJA. In the second series of studies, a limited number of both WT and CF mice were evaluated for safety, toxicity, and efficacy upon oral dosing AJA. As controls, PA infected WT and CF mice were given the vehicle. The mice were followed daily for clinical score and weights for 10 days. At day 10, animals were euthanized and evaluated for bacterial load (colony forming units [CFUs]), total and differential bronchoalveolar lavage (BAL) white blood cell counts (WBCs). In the first study in WT mice, AJA was well tolerated and more efficient at resolving both inflammation and infection than vehicle. CF mice have a more robust inflammatory response to PA infection, and, untreated, are very inefficient at resolving the bacterial burden. Postinfection CF mice lose significant weight and have higher clinical scores. The second study included 4 groups and all animals were chronically infected with PA. All WT animals survived PA infection (both vehicle and drug treated); AJA improved survival of CF mice. Treatment of CF mice with AJA decreased weight loss, BAL WBC counts, and numbers of neutrophils and improved the ability of the animals to resolve pulmonary infection as assessed by lung CFUs. These preliminary data suggest that AJA may be effective in the treatment of inflammation in CF and improve the subject's ability to resolve bacterial infection.

## 3 NONCLINICAL DRUG METABOLISM

### 3.1 Biotransformations of AJA

The in vitro metabolism of AJA by hepatocytes from rats, dogs, cynomolgus monkeys, and humans was studied and the results were reported.^18^ Five metabolites, M1 to M5, were observed in human hepatocyte incubations. One metabolite, M5, a glucuronide, was observed in the chromatogram of canine hepatocyte incubations. In monkey hepatocyte incubations, M5 was observed in the chromatograms of both the 120‐ and 240‐minute samples, a trace metabolite M1 (side‐chain hydroxyl) was observed in the 120‐minute samples, and a trace metabolite M4 (side‐chain dehydrogenation) was observed in the 240‐minute samples. No metabolites were found in the rat hepatocyte incubations. Unchanged amounts of AJA detected after the 2‐hour incubations were 103%, 90%, 86%, and 83% for rat, dog, monkey, and human hepatocytes, respectively.

### 3.2 Lack of effects on cytochrome metabolism of other molecules

Additional studies were done to ascertain if AJA inhibits the activities of 5 of the principal human cytochrome P450 isozymes; (cytochrome P450 isoform 1A2, cytochrome P450 isoform 2C9, cytochrome P450 isoform 2C19, cytochrome P450 isoform 2D6, and cytochrome P450 isoform 3A4/5) CYP1A2, CYP2C9, CYP2C19, CYP2D6, and CYP3A4/5 involved in drug metabolism. In contrast to the phytocannabinoids, THC and CBD, that can inhibit these enzymes, with AJA, no significant inhibition of cytochrome activity was observed. These data further support the conclusion reached in earlier reports on AJA's high margin of safety and indicates that it undergoes minimal metabolism and is not likely to interfere with the normal metabolism of drugs or endogenous substances.

## 4 CLINICAL TRIALS

### 4.1 Pharmacokinetics

As part of a Phase 1 safety trial, the pharmacokinetics of single oral doses of 0‐10 mg of AJA was carried out (Atlantic Pharmaceuticals, unpublished data). A group of 32 healthy adult male subjects was monitored by mass spectrometric methods for 24 hours after dosing. The data showed that AJA was rapidly absorbed and is eliminated with a half‐life of about 3 hours. A linear relationship of the area under the curve (AUC) and *C*_max_ over the 1‐10 mg dose range was observed.

### 4.2 Systemic sclerosis or scleroderma)

Scleroderma, or systemic sclerosis (SSc), is a chronic connective tissue disease generally classified as one of the autoimmune rheumatic diseases. It is estimated that about 90 000 people in the United States and Europe have been diagnosed with SSc. Currently, there is no cure for SSc, but there are several treatments available to relieve specific symptoms. The changes occurring in SSc may affect the connective tissue in many parts of the body. It can involve the skin, esophagus, gastrointestinal tract, lungs, kidneys, heart, and other internal organs. It can also affect blood vessels, muscles, and joints. The tissues of involved organs become hard and fibrous, causing them to function less efficiently. Inflammation is thought to be the driving force behind the disease, leading to increased fibrosis and ultimate mortality. Regarding molecular events, the proinflammatory and profibrotic cytokine TGF‐β has been identified as an important mediator.

A multicenter, double‐blinded, randomized, placebo‐controlled Phase 2 trial to evaluate the safety, tolerability, pharmacokinetics, and efficacy of AJA in subjects with diffuse cutaneous SSc was recently completed (ClinicalTrials.gov; reference Identifier NCT02465437).^1^^,^ 2 Subjects with disease durations of up to 6 years were randomized to receive AJA for the first 4 weeks at 5 mg once a day, 20 mg once a day, 20 mg twice a day, or placebo for the first 4 weeks; subsequently, all drug‐treated subjects received 20 mg twice a day for the next 8 weeks while subjects who had received placebo the first 4 weeks remained on placebo. After 12 weeks, all drug or placebo treatments were stopped and subjects were followed up for another 4 week “washout” period. AJA out‐performed placebo in the American College of Rheumatology Combined Response Index (CRISS) score in diffuse cutaneous SSc. Higher CRISS scores mean greater improvement; a CRISS score ≥20% (CRISS20) is considered a medically meaningful improvement. Differences in categorical levels of CRISS responses and changes from baseline in each of the 5 individual domains of the CRISS score also supported clinical benefit of AJA. The data from this Phase 2 study show that AJA has the potential to provide clinical benefit in patients with systemic sclerosis (SSc).

To corroborate the clinical findings in the SSc trial with quantitative biomarkers, skin biopsies from patients enrolled in the clinical trial were evaluated for changes in gene expression by Whitfield et al^3^ There were significant differences seen between samples from AJA‐treated patients when compared with samples from placebo arms of the study. Extracellular matrix‐related genes important for fibrotic responses and inflammation‐related genes were decreased, whereas lipid metabolism genes important for generating proresolving eicosanoids were increased. These gene expression changes were correlated with AJA‐induced changes in the modified Rodnan skin score, a clinical measure of skin fibrosis.

In April 2016, the sponsor, Corbus Pharmaceuticals, received FDA approval for an open‐label extension to its Phase 2 trial of AJA for SSc.^4^ Subjects entering this open‐label trial will undergo a 28‐day screening period, then receive 20 mg BID AJA for 364 days, ending with a 28‐day washout after the last dose. This study will provide important additional data on long‐term use of AJA in SSc patients.

Recently, Corbus announced FDA approval to move forward with a pivotal Phase 3 clinical trial of AJA in SSc and this trial has now been initiated. This international Phase 3 trial will be a double‐blind, randomized, placebo‐controlled study conducted in ~350 adults with SSc. Subjects will be randomized to receive AJA 20 mg twice per day, AJA 5 mg twice per day, or placebo twice per day. The primary efficacy outcome of the Phase 3 study will be the change from baseline at week 52 in modified Rodnan skin scores (“mRSS”), a measure of skin thickening and a validated clinical outcome in SSc. Secondary outcomes of the Phase 3 study will include the American College of Rheumatology Combined Response Index in Diffuse Cutaneous Systemic Sclerosis (“ACR CRISS”) score, a composite measure of clinical improvement from baseline that incorporates change from baseline in mRSS lung function, and other physician and patient reported outcomes.

### 4.3 Cystic fibrosis

Cystic fibrosis (CF) is a genetic disorder that affects not only the lungs, but also the pancreas, liver, kidneys, and intestine. CF is inherited in an autosomal recessive manner. It is caused by the presence of mutations in both copies of the gene for the CF transmembrane conductance regulator (CFTR) protein. CFTR is involved in production of sweat, digestive fluids, and mucus. When CFTR is not functional, secretions that are usually thin instead become thick. Long‐term issues include difficult breathing and coughing up of mucus as a result of frequent lung infections. Lung infections are treated with antibiotics that may be given intravenously, inhaled, or by mouth. The average life expectancy is between 42 and 50 years in the developed world. Eighty percent of deaths in CF are due to lung problems.

Recently a Phase 2, double‐blinded, randomized, placebo‐controlled multicenter study to evaluate safety, tolerability, and pharmacokinetics of AJA in CF was completed (ClinicalTrials.gov Identifier: NCT02465450) and the topline results have been reported.^18^, ^5^^,^ 6 This trial tested the safety, tolerability, and pharmacokinetics (PK) of AJA in 85 subjects between 18 and 65 years of age with documented CF. An earlier trial in healthy humans measured PK values and is discussed above. There was a screening period of up to 28 days, 84 days of treatment, and 28 days of follow‐up. Primary endpoints were safety and tolerability while secondary endpoints were trends in efficacy (FEV1, Lung Clearance Index, and CFQ‐R Respiratory Symptom Score) and PK. Exploratory endpoints were metabolipidomic profile for MOA, biomarkers of disease activity and inflammation in blood and sputum, and microbiota in the lungs.

This Phase 2 clinical trial met all primary endpoints and showed promising effects in the exploratory endpoints.^18^ Specifically, safety and tolerability were acceptable, not a trivial result considering those have been the main reasons why other anti‐inflammatory drugs (eg, ibuprofen and prednisone) have not been adopted for use in CF despite evidence of efficacy.^19^ Because CF patients have problems absorbing ingested substances, it was important to evaluate the pharmacokinetics of AJA in CF patients. Drug exposures were very similar in the CF patients compared to healthy volunteers indicating that the pharmacokinetics of AJA was not altered. AJA showed evidence of biological activity in the lungs with reduction in multiple inflammatory cells and mediators. Specifically, reductions in neutrophils, eosinophils, lymphocytes, and macrophages were seen in the sputum from AJA‐treated subjects compared to patients receiving placebo, and these effects were dose‐related. Most remarkable, a dose‐related reduction in the rate of pulmonary exacerbations was also noted. Pulmonary exacerbations are believed to have a major detrimental impact on lung function in CF patients. Lung function remained stable throughout the study, and was not expected to improve given the short duration of the trial.

While the study was not powered to establish efficacy, the potential for AJA as an effective inflammation‐resolving agent that can broadly target CF patients, without regard to their specific CFTR gene mutations, was suggested. This is in contrast to other drugs, such as ivacaftor, that target a specific gene and are therefore only effective in a subset of CF patients.^20^

### 4.4 Reduction of inflammation in alveolar macrophages from CF patients

A translational study on the effects of AJA using macrophages from lungs removed from 2 CF patients undergoing lung transplant was recently carried out by Motwani et al^7^ https://doi.org/10.1002/ppul.23837. Cultured macrophages were treated with a lipopolysaccharide derived from *P. aeruginosa*, which is a major bacterial pathogen in CF, to stimulate a cytokine (inflammatory) response. AJA inhibited LPS‐stimulated production of TNF‐α and IL‐6 in a dose‐dependent fashion. Inflammatory cytokine levels are generally elevated in lung macrophages from CF subjects.^21^ Chronic bacterial infection in CF lungs is associated with an excessive inflammatory response, characterized by elevated cytokine levels.

### 4.5 Dermatomyositis

Dermatomyositis (DM) is a connective tissue disease that is characterized by inflammation of the muscles and the skin. It is a systemic disorder that may also affect the joints, the esophagus, the lungs, and the heart. In the United States, the incidence of DM is estimated at 5.5 cases per million people.

An in vitro study using peripheral blood mononuclear cells (PBMCs) from dermatomyositis patients showed that AJA treatment significantly reduced the production of pathogenic cytokines in a dose‐related manner compared with vehicle controls.^22^ Specifically, moderate and high AJA concentrations significantly reduced TNF‐α secretion; all doses significantly decreased the levels of IFN‐α. Cell viability was always greater than 95% following treatment with AJA or vehicle control. These encouraging data supported the initiation of a phase 2 clinical trial.

An NIH supported phase 2 trial of AJA entitled “double‐blind, randomized, placebo‐controlled study to investigate the safety, tolerability, and efficacy in subjects with dermatomyositis” is currently ongoing^8^ (ClinicalTrials.gov Identifier: NCT02466243). The primary outcome measures are: (1) number of participants with treatment emergent adverse events as a measure of safety and tolerability, and (2) change in the cutaneous dermatomyositis disease area and severity index from baseline at 84 days.

The secondary outcome measures are: (1) change in patient‐reported outcomes from baseline at 84 days; (2) change in blood biomarkers of inflammation and disease activity from baseline at 84 days; (3) change in skin biomarkers of inflammation from baseline at 84 days; (4) change in plasma metabolipidomic profiles from baseline at 84 days; and (5) change in AJA plasma concentrations from baseline through 84 days.

The FDA has approved a 1‐year, open‐label extension study of the ongoing phase 2 clinical trial of AJA for the treatment of skin‐predominant dermatomyositis. The goal of the open‐label study is to collect long‐term safety and efficacy data on AJA. The safety and efficacy endpoints used in the double‐blinded, placebo‐controlled portion of the study will be measured throughout the 1‐year extension study.

## 5 SAFETY AND TOLERABILITY

### 5.1 Lack of ulcerogenicity

The most significant unwanted side effect of many anti‐inflammatory agents is the formation of gastrointestinal ulcers. For this reason, AJA was carefully examined to detect any possible ulcerogenicity. When given acutely to rats in doses up to 1000 mg/kg, no evidence for ulcer formation was seen. Chronic intragastric administration of up to 30 mg/kg likewise resulted in no ulcerogenicity, whereas the positive control indomethacin‐treated rats showed extensive ulcer formation.^23^ AJA has a very favorable therapeutic index for ulcerogenicity vs inflammation when compared to several NSAIDs (Table 1).

**Table 1:** Therapeutic index of AJA compared with selected NSAIDs^a^

Drug	Therapeutic index
Ajulemic acid	>300
Ketorolac	7.28
Ketoprofen	41.0
Indomethacin	13.3
Diclofenac	13.8
Ibuprofen	3.59

AJA, ajulemic acid; NSAID, nonsteroidal anti‐inflammatory drug.

a The therapeutic indices were calculated from the ratio of the ED_50_ ulcerogenicity/ED‐50 adjuvant‐induced arthritis in the rat (95% confidence limits). The ED_50_ values for AJA are estimates since a dose of up to 30 mg/kg/day did not produce detectable ulceration (E. Dajani, unpublished data). The ED_50_ in the rat adjuvant arthritis test for AJA is estimated.^3^

### 5.2 Toxicity

Because of its structural similarity to THC, AJA was studied for possible induction of opiate like physical dependence in a 14‐day rat study.^24^ None of the typical opiate withdrawal effects such as writhing, diarrhea, wet dog shakes, etc., were observed indicating that AJA has a low dependence liability. There were no effects on renal, cardiovascular, or gastrointestinal function, and no signs of respiratory depression as well. Lethal doses were estimated following single doses in mice (600 mg/kg) and in rats (400 mg/kg). AJA was well tolerated at doses up to 50 mg/kg. Three different standard tests for mutagenic potential gave negative findings.

## 6 MECHANISM OF ACTION

### 6.1 Cannabinoid receptors

In recent years, the discovery of CB2‐specific agonists has been an important goal in the area of therapeutics. CB2 is a member of the G‐protein‐coupled receptor (GPCR) superfamily and its pharmacology has been reviewed in some detail.^25^ CB2 receptors are primarily expressed in the periphery, including cells involved in immune system activities. Although CB2 has been detected in the CNS, its actions do not include mood alteration or behavioral responses. Receptor binding studies using highly purified preparations of AJA showed ~12‐fold selectivity for CB2 over CB1 receptors, whereas preparations described in previous reports showed much higher activity at CB1 relative to CB2 (Table 2). The highly purified preparation of AJA stimulated release of PGJ in a CB2‐dependent manner and produced minimal catalepsy or hypothermia, effects primarily mediated by CB1 receptors.^2^ In contrast, AJA preparations described in earlier reports had higher CB1 binding activity and caused concomitantly greater catalepsy and hypothermia,^2^ suggesting possible contamination by impurities such as 11‐hydroxy‐DMH‐THC, an intermediate in the synthesis of AJA.

**Table 2:** Cannabinoid receptor binding data for several ajulemic acid preparations^a^

Ligand	Ki CB1	Ki CB2	CB1/CB2	Reference
Ultrapure AJA (JBT‐101)	628	51	12.3	Tepper et al^32^
Ajulemic acid	5.7	56.1	0.10	Dyson et al^26^
Ajulemic acid	32.3	170.5	0.19	Rhee et al^33^
WIN‐55,212^b^	1.89‐123	0.28‐16.2	ND	Pertwee et al^34^
SR144528^b^	50.3‐10 000	0.28‐5.6	ND	Pertwee et al^34^
SR141716^b^	1.8‐12.3	514‐13 200	ND	Pertwee et al^34^

AJA, ajulemic acid.

a All Ki values are expressed in nmol/L units of concentration.

b Included for comparison with AJA. SR144528 is a widely used CB2 antagonist. SR141716 is a CB1 antagonist also called Rimonabant.

### 6.2 Drug distribution

The structure of AJA suggests an inability to cross the blood‐brain barrier in humans due to its polarity and molecular weight. Evidence from distribution studies in rats is in agreement with this possibility.^26^ Therefore, in addition to its selectivity for CB2 over CB1, the poor brain uptake of AJA will further favor its actions toward activating CB2 receptors on immune cells in the periphery, as opposed to stimulating CB1 receptors in the CNS.

### 6.3 Preclinical mechanism studies

**Figure 3:**
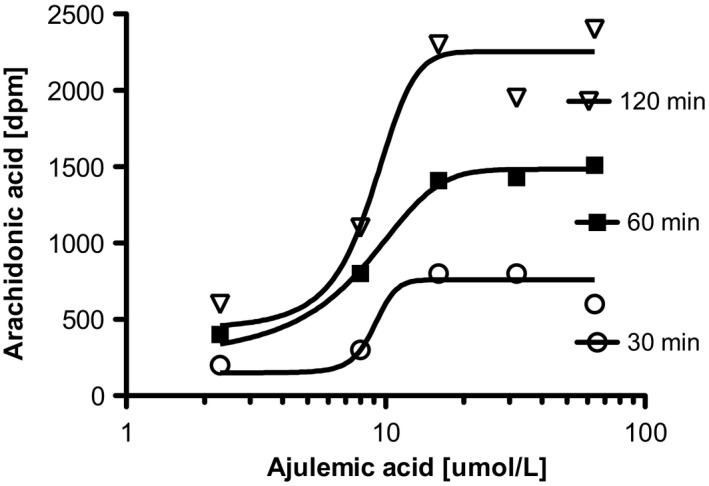
AJA induces release of free arachidonic acid in C6 glioma cells (Burstein SH, unpublished data). Cells were labeled for 20 hour with ^14^C‐arachidonic acid, media (RPMI + 0.1% BSA) were changed and cells were treated for indicated times with AJA in 10 μL of DMSO. The control was 10 μL of DMSO. Release was measured by liquid scintillation counting on a 0.1 mL aliquot of medium. Values shown are means ± SD. N = 4. AJA, ajulemic acid

**Figure 4:**
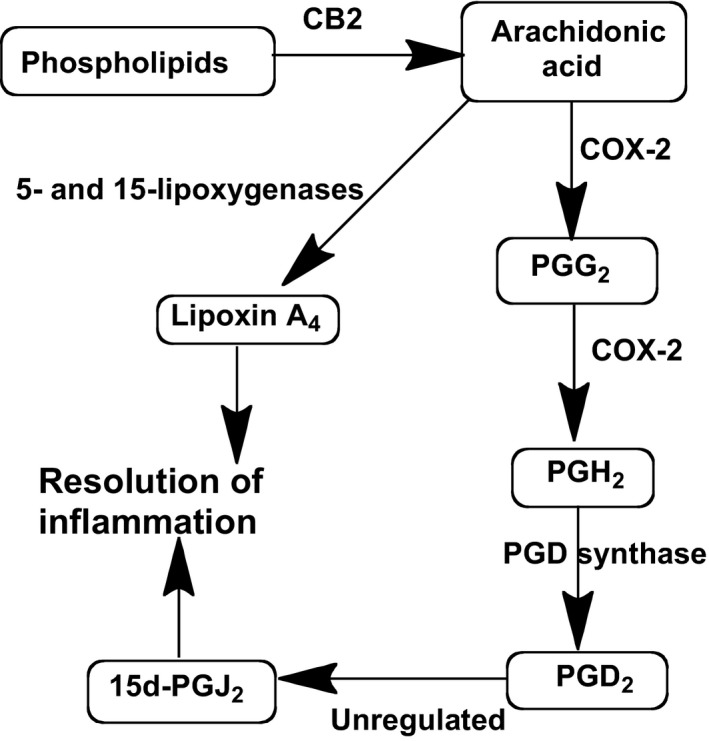
In vitro CB2‐initiated pathways for the synthesis of the proresolving eicosanoids PGJ_2_ and LXA_4_. All of the steps are tightly regulated except for the conversion of PGD_2_ to PGJ_2_, which proceeds in the absence of enzymic mediation. PGD_2_, prostaglandin D_2_; LXA_4_, lipoxin A_4_

The major precursor for all of the eicosanoids is free arachidonic acid (AA) whose release from phospholipid storage sites is robustly stimulated by AJA (Figure 3).^27^ This initiates a cascade of events leading to the eicosanoid superfamily, one branch of which is shown in Figure 4. Several members of the eicosanoid superfamily shown in Figure 4 have been implicated in the events that regulate the resolution of chronic inflammation.^28^ A group of these known as the cyclopentenone prostaglandins are the result of nonenzymically mediated conversions of prostaglandin D_2_ (PGD_2_), which is a beta‐hydroxy ketone to the PGJ series. As could be expected, the resultant alpha, beta‐unsaturated ketone is subject to possible Michael addition reactions with various molecules that may be available. The terminal product of PGD_2_ metabolism is 15‐deoxy‐Δ^12,14^‐prostaglandin‐J_2_ (PGJ). PGJ has been shown to suppress various proinflammatory signaling pathways such as those that function through NF‐kB, AP1, signal transducers and activators of transcription. PGJ also causes suppression of inducible nitric oxide synthase, interleukin‐1α, TNF‐β, and IL‐12.

Regulation of eicosanoids involved in resolution of inflammation by AJA may occur through CB2 and at several other places within the cascade shown in Figure 4. The bifunctional enzyme COX‐2 oxidizes AA to PGG_2_ and a subsequent peroxidase reaction leads to PGH_2_. Then, mediated by the terminal PGD synthase, PGD_2_ is formed, which in turn is transformed to the PGJ series in an unregulated manner. In addition to stimulating production of AA, AJA stimulates COX‐2 activity, and has a selective effect on PGD synthase activity. All of this results in an increase in levels of the proresolving PGJ, however, there is little change in the level of the proinflammatory prostaglandin PGE_2_ (data not shown).

The ability of AJA to increase the production of the proresolving eicosanoid PGJ has been confirmed experimentally; application of AJA to HL60 cells produced a robust stimulation of PGJ.^27^ The AJA‐induced increase in PGJ was effectively inhibited by the CB2 antagonist SR144528 (1 μmol/L), whereas the CB1 antagonist SR141716 (10 μmol/L) had only a small effect, suggesting a CB2 receptor‐mediated mechanism of action.^1^, ^27^

A second eicosanoid, lipoxin A_4_ (LXA_4_), has also been reported to contribute to the inflammation resolving action of AJA.^29^ In a different branch of the arachidonic acid cascade, LXA_4_ production results from the sequential actions of 5‐ and 15‐lipoxygenases (LOX) following the release of free arachidonic acid (Figure 4).^29^ It was further shown that the 12/15 LOX inhibitor baicalein reduces this action of AJA. LXA_4_ is an important member of the emerging class of molecules known as specialized proresolving mediators (SPMs) that promote the resolution of chronic inflammation.^30^ Following trauma or tissue injury, SPMs play pivotal roles in vascular responses and leukocyte trafficking, from initiation to resolution. Organ fibrosis can be an ultimate result of unresolved chronic inflammation.

### 6.4 Mechanism studies in humans

To further explore its mechanism of action, and establish a pharmacodynamic relationship between dose and effect, ultrapure AJA, was evaluated in a model of experimental inflammation in humans. This model utilizes intradermal injection of UV‐killed *Escherichia coli* into the forearm of healthy volunteers.^31^ Inflammation was detected by increased blood flow, neutrophilia, and increased levels of proinflammatory cytokines. Resolution was observed by a decrease in blood flow, a reduction in neutrophils, an increase in monocytes/macrophages, and a drop of classic proinflammatory cytokine levels. It was claimed that this model can provide mechanistic insights and can help evaluate the clinical potential of novel anti‐inflammatory and proresolving drugs.

AJA was studied in the above model and preliminary data were reported by Gilroy et al^9^ Subjects were divided into 3 groups of 5, each receiving 5 mg or 20 mg BID AJA, or placebo, all of which were given before the procedure. Both doses of AJA caused a 70% reduction in inflammation based on a decrease in neutrophil infiltration and decreased blood flow around the site of inflammation. In addition, the treatment resulted in a progressive increase in blood flow around the site of inflammation during the early phases of resolution indicative of an efficient inflammatory response followed by a timely resolution^9^.

## 7 SUMMARY AND CONCLUSIONS

Thus far, the available preclinical (Table 3) and clinical data (Table 4) suggest that AJA could provide a safe and effective treatment for diseases that are characterized by chronic inflammation and eventually result in fibrosis, loss of function, etc. Data from in vitro experiments suggest that the CB2 receptor is an important mediator and that AJA can act as an inflammation‐resolving agent through its stimulatory action on the production of the proresolving agents PGJ and LXA_4_. Its ability to lower IL‐1β inhibitor a factor in some of its actions. Based on its structural overlap with AA, it has been suggested that AJA should be referred to as an *arachidomimetic* agent.^1^ Therapeutic targets for AJA such as scleroderma, CF, dermatomyositis, lupus, arthritis, MS, and metastatic cancer are possibilities based on the current body of data. Its lack of adverse side effects makes it especially suitable for chronic administration. Finally, a long‐standing goal in the cannabinoid field has been the separation of therapeutic actions of THC from its psychoactivity. AJA appears to represent, in a large measure, such an achievement of this goal.

**Table 3:** Reported preclinical anti‐inflammatory and antifibrotic actions of ajulemic acid

Observed action	Model	Reference
Reduces paw edema in mice	Arachidonate or PAF induction	Burstein et al^35^
Reduces leukocyte adhesion	Mouse peritoneal cells	Burstein et al^35^
Decreases joint damage	Adjuvant‐induced arthritis in rats	Zurier et al^3^
Reduces leukocyte migration	Subcutaneous air pouch	Zurier et al^3^
Reduces MS‐induced spasticity	EAE mouse model	Pryce et al^6^
Antimetastatic Effects	Mouse glioma in vivo	Recht L et al, unpublished.
Reduction of IL‐1β levels	Blood and synovial monocytes	Zurier et al^12^
Inhibits matrix metalloproteinases	Human synovial cells	Johnson et al^14^
Inhibits cell proliferation in vitro	Several cancer cell lines	Recht et al^9^
Apoptosis of human T cells	Caspase‐3 activity	Bidinger et al^4^
Potent antifibrotic action^a^	Bleomycin in mice	Gonzalez et al^7^, Lucattelli et al^8^
Pathogen resolution^a^	CFTR‐deficient mice	Bonfield et al^16^
Reduced peritoneal cell infiltration	Zymosan‐induced peritonitis	Zurier et al^29^
Antichemical blister activity	Human keratinocyte cell cultures	Atlantic Pharmaceuticals, unpublished

AJA, ajulemic acid; CFTR, CF transmembrane conductance regulator.

a The ultrapure preparation of AJA (JBT‐101) with reduced CB1 activity was used for these studies.^32^

**Table 4:** Ongoing and planned Phase 2 clinical trials

Target	Patient population	Sample size	Doses	Key findings
Systemic sclerosis (SSc)	Adults with active diffuse cutaneous SSc	41	5, 10 & 20 mg	71% median of subjects achieved improvement in CRISS
Cystic fibrosis (CF)	CF patients, 18‐65 years	70	5 mg/20 mg bid^a^	Reduces acute pulmonary exacerbations and multiple inflammatory biomarkers
Dermatomyositis	Adults with DM	22	20 mg bid	Improved CDASI by 9.3 vs 3.7 for placebo; *P *=* *.04^b^
Systemic lupus erythematosus (SLE)	Adults with SLE	100	5 mg	Efficacy in inflammatory pain^c^

CRISS, American College of Rheumatology Combined Response Index Systemic Sclerosis**.**

a 5 mg once a day on Days 1‐28, then 20 mg twice a day on Days 29‐84.

b Cutaneous Dermatomyositis Disease Area and Severity Index (CDASI).

c Planned to begin first quarter 2018.

## AUTHORSHIP CONTRIBUTIONS

Sumner Burstein wrote the manuscript.

## DISCLOSURES

The author is the discoverer of ajulemic acid and is a cofounder of Corbus Pharmaceuticals, Inc.

## Endnotes

1 Man A, Dgetluck N, White B. Prospective validation of the Systemic Sclerosis Skin Symptoms Patient‐Reported Outcome (SSPRO) in a phase 2 trial of anabasum (JBT‐101) in diffuse cutaneous systemic sclerosis (dcSSc) [abstract]. *Arthritis Rheumatol*. 2017;69(suppl 10). http://acrabstracts.org/abstract/prospective-validation-of-the-systemic-sclerosis-skin-symptoms-patient-reported-outcome-sspro-in-a-phase-2-trial-of-anabasum-jbt-101-in-diffuse-cutaneous-systemic-sclerosis-dcssc/. Accessed September 20, 2017.

2 Spiera RF, Hummers LK, Chung L, Frech TM, Domsic RT, Hsu V, Furst DE, Gordon JK, Mayes MD, Simms RW, Constantine S, White B. A phase 2 study of safety and efficacy of anabasum (JBT‐101), a cannabinoid receptor type 2 agonist, in diffuse cutaneous systemic sclerosis [abstract]. *Arthritis Rheumatol*. 2017;69(suppl 10). http://acrabstracts.org/abstract/a-phase-2-study-of-safety-and-efficacy-of-anabasum-jbt-101-a-cannabinoid-receptor-type-2-agonist-in-diffuse-cutaneous-systemic-sclerosis/. Accessed September 22, 2017.

3 Martyanov V, Nesbeth Y, Cai G, Wood TA, Reder J, Constantine S, White B, Spiera RF, Whitfield ML. Effect of anabasum (JBT‐101) on gene expression in skin biopsies from subjects with diffuse cutaneous systemic sclerosis (dcSSc) and the relationship of baseline molecular subsets to clinical benefit in the phase 2 trial [abstract]. *Arthritis Rheumatol*. 2017;69(suppl 10). http://acrabstracts.org/abstract/effect-of-anabasum-jbt-101-on-gene-expression-in-skin-biopsies-from-subjects-with-diffuse-cutaneous-systemic-sclerosis-dcssc-and-the-relationship-of-baseline-molecular-subsets-to-clinical-benefit/. Accessed September 20, 2017.

4 Spiera RF, Hummers LK, Chung L, Frech TM, Domsic RT, Hsu V, Furst DE, Gordon JK, Mayes MD, Simms RW, Constantine S, White B. safety and efficacy of anabasum (JBT‐101) in diffuse cutaneous systemic sclerosis (dcSSc) subjects treated in an open‐label extension of trial JBT101‐SSc‐001 [abstract]. *Arthritis Rheumatol*. 2017;69(suppl 10). http://acrabstracts.org/abstract/safety-and-efficacy-of-anabasum-jbt-101-in-diffuse-cutaneous-systemic-sclerosis-dcssc-subjects-treated-in-an-open-label-extension-of-trial-jbt101-ssc-001/. Accessed September 20, 2017.

5 Ribeiro CM. Zhang G, Lubamba BA, Tepper M. Anabasum reduces excessive inflammatory responses in cystic fibrosis patient‐derived lung macrophages. Poster No. 104: *Pediatr Pulmonol*. 2017;52:S1‐S82. https://doi.org/10.1002/ppul.23837.

6 Chmiel J, Elborn S, Constantine S, White B. A double‐blind, placebo controlled phase 2 study in adults with cystic fibrosis of anabasum, a selective cannabinoid receptor type 2 agonist. Poster No. 272: *Pediatr Pulmonol*. 2017;52: S1‐S82. https://doi.org/10.1002/ppul.23837.

7 Motwani M, Bennett F, Gilroy D, Tepper M, White, B. Poster No. 312: anabasum enhances resolution of bacterial‐induced inflammation in healthy humans; *Pediatr Pulmonol*. 2017;52:S1‐S82.

8 Werth VP, Hejazi E, Pena SM, Haber JS, Okawa J, Feng R, Gabre K, Concha J, Constantine S, White B. Comparison of patients with dermatomyositis in a specialty clinic versus clinical trial with anabasum (JBT‐101), a cannabinoid receptor type 2 agonist [abstract]. *Arthritis Rheumatol*. 2017;69(suppl 10). http://acrabstracts.org/abstract/comparison-of-patients-with-dermatomyositis-in-a-specialty-clinic-versus-clinical-trial-with-anabasum-jbt-101-a-cannabinoid-receptor-type-2-agonist/. Accessed September 20, 2017.

9 Motwani M, Bennett F, Tepper M, White B, Norris P, MacAllister R, Serhan C, Gilroy D. Anabasum (JBT‐101) enhances resolution of inflammation in humans [abstract]. *Arthritis Rheumatol*. 2017; 69 (suppl 10). http://acrabstracts.org/abstract/anabasum-jbt-101-enhances-resolution-of-inflammation-in-humans/. Accessed September 20, 2017.

## References

[1] Burstein SH . The cannabinoid acids, analogs and endogenous counterparts. Bioorg Med Chem. 2014;22:2830–2843. 24731541 10.1016/j.bmc.2014.03.038 PMC4351512

[2] Zurier RB , Burstein SH . Cannabinoids, inflammation, and fibrosis. FASEB J. 2016;30:3682–3689. 27435265 10.1096/fj.201600646R

[3] Zurier RB , Rossetti RG , Lane JH , Goldberg JM , Hunter SA , Burstein SH . Dimethylheptyl‐THC‐11 oic acid: a nonpsychoactive antiinflammatory agent with a cannabinoid template structure. Arthritis Rheum. 1998;41:163–170. 9433882 10.1002/1529-0131(199801)41:1<163::AID-ART20>3.0.CO;2-9

[4] Bidinger B , Torres R , Rossetti RG , et al. Ajulemic acid, a nonpsychoactive cannabinoid acid, induces apoptosis in human T lymphocytes. Clin Immunol. 2003;108:95–102. 12921755 10.1016/s1521-6616(03)00064-0

[5] George KL , Saltman LH , Stein GS , Lian JB , Zurier RB . Ajulemic acid, a nonpsychoactive cannabinoid acid, suppresses osteoclastogenesis in mononuclear precursor cells and induces apoptosis in mature osteoclast‐like cells. J Cell Physiol. 2008;214:714–720. 17786950 10.1002/jcp.21263

[6] Pryce G , Visintin C , Ramagopalan SV , et al. Control of spasticity in a multiple sclerosis model using central nervous system‐excluded CB1 cannabinoid receptor agonists. FASEB J. 2014;28:117–130. 24121462 10.1096/fj.13-239442

[7] Gonzalez EG , Selvi E , Balistreri E , et al. Synthetic cannabinoid ajulemic acid exerts potent antifibrotic effects in experimental models of systemic sclerosis. Ann Rheum Dis. 2012;71:1545–1551. 22492781 10.1136/annrheumdis-2011-200314

[8] Lucattelli M , Fineschi S , Selvi E , et al. Ajulemic acid exerts potent anti‐fibrotic effect during the fibrogenic phase of bleomycin lung. Respir Res. 2016;17:49. 27153807 10.1186/s12931-016-0373-0 PMC4859981

[9] Recht LD , Salmonsen R , Rosetti R , et al. Antitumor effects of ajulemic acid (CT3), a synthetic non‐psychoactive cannabinoid. Biochem Pharmacol. 2001;62:755–763. 11551521 10.1016/s0006-2952(01)00700-6

[10] Kim S , Karin M . Role of TLR2‐dependent inflammation in metastatic progression. Ann N Y Acad Sci. 2011;1217:191–206. 21276007 10.1111/j.1749-6632.2010.05882.x PMC4383094

[11] Apte RN , Dotan S , Elkabets M , et al. The involvement of IL‐1 in tumorigenesis, tumor invasiveness, metastasis and tumor‐host interactions. Cancer Metastasis Rev. 2006;25:387–408. 17043764 10.1007/s10555-006-9004-4

[12] Zurier RB , Rossetti RG , Burstein SH , Bidinger B . Suppression of human monocyte interleukin‐1beta production by ajulemic acid, a nonpsychoactive cannabinoid. Biochem Pharmacol. 2003;65:649–655. 12566094 10.1016/s0006-2952(02)01604-0

[13] Ridker P . Effect of interleukin‐1β inhibition with canakinumab on incident lung cancer in patients with atherosclerosis: exploratory results from a randomised, double‐blind, placebo‐controlled trial. Lancet. 2017;390:1833–1842. 28855077 10.1016/S0140-6736(17)32247-X

[14] Johnson DR , Stebulis JA , Rossetti RG , Burstein SH , Zurier RB . Suppression of fibroblast metalloproteinases by ajulemic acid, a nonpsychoactive cannabinoid acid. J Cell Biochem. 2007;100:184–190. 16927387 10.1002/jcb.21046

[15] Cowan FM , Broomfield CA . Putative roles of inflammation in the dermatopathology of sulfur mustard. Cell Biol Toxicol. 1993;9:201–213. 8299000 10.1007/BF00755599

[16] Bonfield TL , Tepper MA . Cystic Fibrosis Foundation Research Conference: Pushing the Frontiers. 2015 https://d1io3yog0oux5.cloudfront.net/_9a1d7997e95b56a407e2442b025d1f17/corbuspharma/db/228/2110/pdf/Corbus+CFF+Frontiers+Poster+052915vf.pdf (Last access March 2018)

[17] Burstein SH , Tepper MA . In vitro metabolism and metabolic effects of ajulemic acid, a synthetic cannabinoid agonist. Pharmacol Res Perspect. 2013;1:e00017. 25505570 10.1002/prp2.17 PMC4186433

[18] Chmiel JF , Elborn JS , Constantine S , White B . A Phase 2 study of the safety, pharmacokinetics, and efficacy of anabasum (JBT‐101) in cystic fibrosis (CF). J Cyst Fibros. 2017;16S1:S01.05.

[19] Konstan MW , Byard PJ , Hoppel CL , Davis PB . Effect of high‐dose ibuprofen in patients with cystic fibrosis. N Engl J Med. 1995;332:848–854. 7503838 10.1056/NEJM199503303321303

[20] Ramsey BW , Davies J , McElvaney NG , et al. Group VXS . A CFTR potentiator in patients with cystic fibrosis and the G551D mutation. N Engl J Med. 2011;365:1663–1672. 22047557 10.1056/NEJMoa1105185 PMC3230303

[21] Bruscia EM , Zhang PX , Ferreira E , et al. Macrophages directly contribute to the exaggerated inflammatory response in cystic fibrosis transmembrane conductance regulator‐/‐ mice. Am J Respir Cell Mol Biol. 2009;40:295–304. 18776130 10.1165/rcmb.2008-0170OC PMC2645527

[22] Robinson ES , Alves P , Bashir MM , Zeidi M , Feng R , Werth VP . Cannabinoid reduces inflammatory cytokines, tumor necrosis factor‐alpha, and type I interferons in dermatomyositis in vitro. J Invest Dermatol. 2017;137:2445–2447. 28652111 10.1016/j.jid.2017.05.035 PMC5651184

[23] Dajani EZ , Larsen KR , Taylor J , et al. 1′,1′‐Dimethylheptyl‐delta‐8‐tetrahydrocannabinol‐11‐oic acid: a novel, orally effective cannabinoid with analgesic and anti‐inflammatory properties. J Pharmacol Exp Ther. 1999;291:31–38. 10490883

[24] Burstein S . Ajulemic acid (IP‐751): synthesis, proof of principle, toxicity studies, and clinical trials. AAPS J. 2005;7:E143–E148. 16146336 10.1208/aapsj070115 PMC2751505

[25] Han S , Thatte J , Buzard DJ , Jones RM . Therapeutic utility of cannabinoid receptor type 2 (CB(2)) selective agonists. J Med Chem. 2013;56:8224–8256. 23865723 10.1021/jm4005626

[26] Dyson A , Peacock M , Chen A , et al. Antihyperalgesic properties of the cannabinoid CT‐3 in chronic neuropathic and inflammatory pain states in the rat. Pain. 2005;116:129–137. 15936883 10.1016/j.pain.2005.03.037

[27] Stebulis JA , Johnson DR , Rossetti RG , Burstein SH , Zurier RB . Ajulemic acid, a synthetic cannabinoid acid, induces an antiinflammatory profile of eicosanoids in human synovial cells. Life Sci. 2008;83:666–670. 18840450 10.1016/j.lfs.2008.09.004

[28] Gilroy DW . Eicosanoids and the endogenous control of acute inflammatory resolution. Int J Biochem Cell Biol. 2010;42:524–528. 20026423 10.1016/j.biocel.2009.12.013

[29] Zurier RB , Sun YP , George KL , et al. Ajulemic acid, a synthetic cannabinoid, increases formation of the endogenous proresolving and anti‐inflammatory eicosanoid, lipoxin A4. FASEB J. 2009;23:1503–1509. 19124557 10.1096/fj.08-118323 PMC2669421

[30] Serhan CN . Pro‐resolving lipid mediators are leads for resolution physiology. Nature. 2014;510:92–101. 24899309 10.1038/nature13479 PMC4263681

[31] Motwani MP , Flint JD , De Maeyer RP , et al. Novel translational model of resolving inflammation triggered by UV‐killed E. coli. J Pathol Clin Res. 2016;2:154–165. 27499924 10.1002/cjp2.43 PMC4958736

[32] Tepper MA , Zurier RB , Burstein SH . Ultrapure ajulemic acid has improved CB2 selectivity with reduced CB1 activity. Bioorg Med Chem. 2014;22:3245–3251. 24856183 10.1016/j.bmc.2014.04.062

[33] Rhee MH , Vogel Z , Barg J , et al. Cannabinol derivatives: binding to cannabinoid receptors and inhibition of adenylylcyclase. J Med Chem. 1997;40:3228–3233. 9379442 10.1021/jm970126f

[34] Pertwee RG , Howlett AC , Abood ME , et al. International Union of Basic and Clinical Pharmacology. LXXIX. Cannabinoid receptors and their ligands: beyond CB(1) and CB(2). Pharmacol Rev. 2010;62:588–631. 21079038 10.1124/pr.110.003004 PMC2993256

[35] Burstein SH , Audette CA , Breuer A , et al. Synthetic nonpsychotropic cannabinoids with potent antiinflammatory, analgesic, and leukocyte antiadhesion activities. J Med Chem. 1992;35:3135–3141. 1507202 10.1021/jm00095a007

